# Leptin, CRP, and adiponectin correlate with body fat percentage in adolescents: systematic review and meta-analysis

**DOI:** 10.3389/fnut.2025.1560080

**Published:** 2025-07-11

**Authors:** Ariane Ribeiro de Freitas Rocha, Nubia de Souza de Morais, Francilene Maria Azevedo, Dayane de Castro Morais, Patrícia Feliciano Pereira, Maria do Carmo Gouveia Peluzio, Sylvia do Carmo Castro Franceschini, Silvia Eloiza Priore

**Affiliations:** Department of Nutrition and Health, Federal University of Viçosa, Viçosa, Brazil

**Keywords:** adipose tissue, adolescence, body composition, inflammatory markers, cardiometabolic risk

## Abstract

**Introduction:**

Adipose tissue is important in the secretion of inflammatory substances, and may be directly or indirectly associated to the development of cardiovascular and metabolic diseases in adolescence.

**Objective:**

To evaluate whether inflammatory markers are associated to body fat percentage in adolescents.

**Methodology:**

Systematic review conducted following the items of the PRISMA, and registered in PROSPERO. The descriptors adolescent, body fat distribution and cytokines were combined together in the electronic databases: Scopus, PubMed, Embase, Cochrane, Scholar Google and ProQuest, independently by two researchers, in January 2022 and atualized in November 2024. Meta-analysis of the correlation of inflammatory markers with body fat percentage was conducted using the metabin function of the meta package of the RStudio software (4.0.4).

**Results:**

Resulted in 7,592 records, of which 31 articles met the inclusion criteria and were selected for this study. Cross-sectional and prospective cohort observational studies were included. The meta-analysis included 4,682 adolescents, aged 10 to 19 years, of both sexes. The inflammatory markers leptin and C-reactive protein were positively correlated (*r* = 0.67; *r* = 0.32) and adiponectin was negatively correlated (*r* = −0.23) with body fat percentage in adolescents of both sexes.

**Conclusion:**

In adolescents, the body fat percentage is related to the inflammatory markers leptin, C-reactive protein and adiponectin. It is important to evaluate the body fat composition of adolescents in clinical practice to identify those with a higher percentage of fat, that may reflect an inflammatory profile, as well as increased cardiometabolic risk that accompanies adolescents into adulthood.

**Systematic review registration:**

https://www.crd.york.ac.uk/PROSPERO/view/CRD42020208305, identifier PROSPERO: CRD42020208305.

## Graphical Abstract

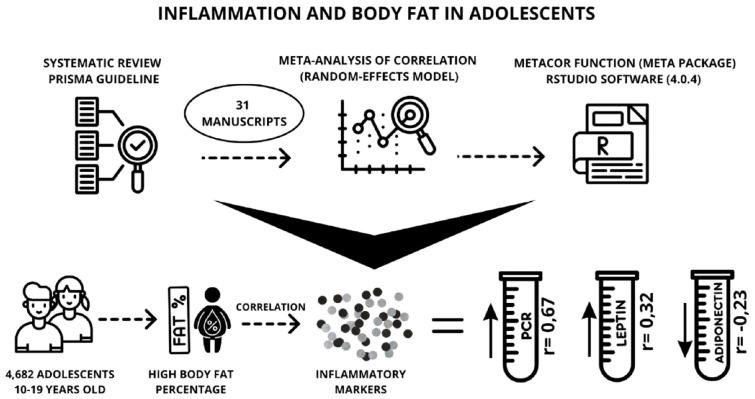

## Introduction

Adolescence is the chronological period between the ages of 10 and 19 years, which includes important physical and psychosocial changes that lead to grow and develop (1, 2). Behavioral changes during this period can promote unhealthy habits, such as a sedentary lifestyle and poor diet, which, together with genetic and physiological components, are risk factors for overweight and obesity (3–5).

Obesity has affected adolescents all over the world. It is the most prevalente chronic non-communicable disease in these individuals (6). Body mass index (BMI) is the most widely used indicator to identify obesity, but it is unable to differentiate adipose tissue from fat-free tissue, and can significantly underestimate the prevalence of obesity. For adolescents, it has the important limitation of not using cut-off points established on the basis of prognosis of morbidity or mortality, unlike adults. In addition, the BMI cutoff points in adolescents are not correlated with the biological outcomes and do not take into account the association between sexual maturation and anthropometric measurements (3). Therefore, it is necessary to assess the body fat composition of adolescents, since percentages equal to or greater than 20% in males and 25% in females are associated with high cardiometabolic risk in these individuals (7). In addition, adolescents classified as having a normal BMI may have a body fat percentage (%BF) above normal and be at a similar risk to those with obesity (high BMI and high %BF) of developing cardiometabolic diseases (8–14).

The production and secretion of inflammatory cytokines tends to be higher in adults with a high body fat percentage (15) because adipose tissue, in addition to storing energy, regulating temperature and providing mechanical protection, is a dynamic organ that secretes pro-inflammatory and anti-inflammatory, involved in the process of subclinical inflammation (16). Excess adipose tissue can both release direct markers of inflammation, such as adiponectins and leptins, as well as cytokines involved in the synthesis of other markers, such as adipocytes involved in the stimulation of hepatic C-reactive protein (CRP) synthesis. Thus, the increase in adipose tissue results in macrophage infiltration and increased levels of inflammatory substances in the tissue and circulation, which are directly or indirectly associated to the development of cardiovascular and metabolic diseases in these individuals (17, 18).

It remains to be seen whether this also applies to adolescents, mainly because we believe that at this stage of life, the higher the percentage of body fat, the higher the levels of inflammatory markers. And that this inflammation may be present even before the development of cardiometabolic complications, being an important risk factor that can continue or worsen in adult life. In this sense, the objective of this review was to evaluate whether inflammatory markers are associated to body fat percentage in adolescents.

## Methodology

### Data source and research strategy

This systematic review was conducted following the items of the Preferred Reporting Items for Systematic Reviews – PRISMA (19) and registered with PROSPERO under number CRD42020208305.

The Population, Exposure, Comparator, and Outcomes (PECOS) method was adopted for the inclusion criteria, to answer whether inflammatory markers are associated with body fat percentage in adolescents. The population consisted of adolescents aged 10–19 years; exposure was body fat percentage; there was no comparison; the expected outcome was the presence of inflammatory markers; and cross-sectional and prospective cohort studies were included.

The research considered all inflammatory markers related to body fat in adolescents, secreted or not by adipocytes, in order to verify the role of body fat in the development of subclinical inflammation in the body. Moreover, uric acid, even not being characterized as a marker of inflammation, was also included in this review because, although its important role as an antioxidant in the circulation is known, in increased levels, such as above 5 mg/dL. In addition, uric acid, although not characterized as an inflammation marker, was also included in this review because, although its important role as an antioxidant in the circulation is known, at increased levels, such as above 5 and 8 mg/dL (20) for women and men, respectively, it is strongly related to metabolic complications associated with fat tissue.

All methods of body fat assessment were considered to obtain a larger number of studies, since in the published studies there is high heterogeneity in the choice of methods.

The search for articles was carried out in January 2022, and update in November 2024, in the electronic databases: Scopus, PubMed, Embase, Cochrane, Scholar Google and ProQuest. Rayyan bibliographic software was used to store the references. The steps were performed without a filter, using different combinations of the Medical Subject Headings (MeSH) and Health Sciences Descriptors (DecS), namely: adolescent, body fat distribution and cytokines. The complete search strategy is available in Supplementary material—Appendix I.

### Selection of studies

**FIGURE 1:**
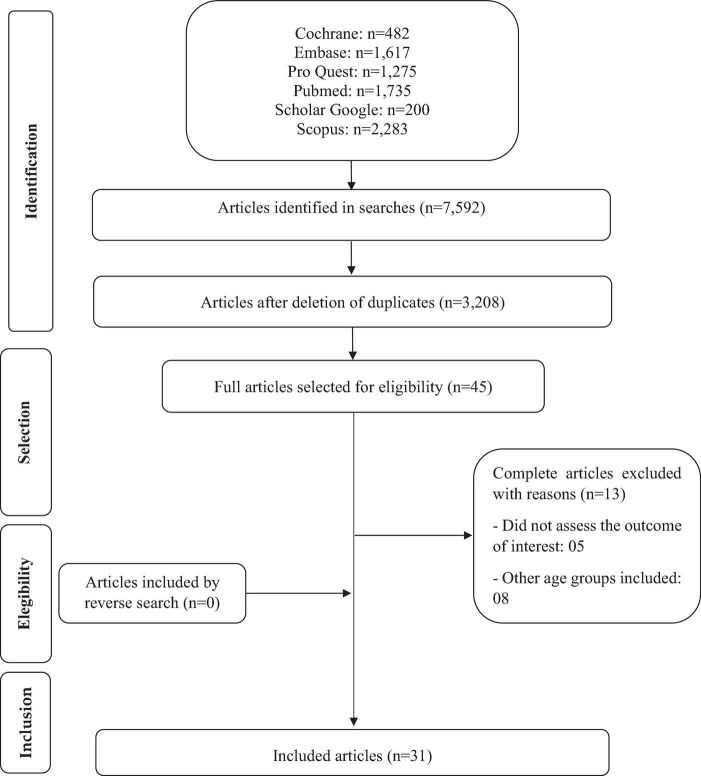
Flowchart of articles selected in the systematic review.

The steps for the search (Figure 1) were performed independently by two reviewers, using the Rayyan software (Qatar Computing Research Institute, Qatar). A search for articles was carried out in the databases, checking the total number found; exclusion of duplicate; title reading for initial selection; subsequent selection for reading the abstracts. Finally, the complete analysis to verify which studies met the eligibility criteria. The included and excluded studies were determined by consensus among the reviewers. A reverse search was performed, which consists of analyzing the references of the selected articles to identify studies that were not included in the databases, but no study of interest was found.

### Eligibility criteria

Original articles from studies that evaluated the correlation between the presence of inflammatory markers and body fat percentage in adolescents aged 10–19 years were included. To ensure greater scope in the research, there was no language distinction or restriction on the time of publication of the studies. Studies involving adolescents undergoing surgery, such as stomach reduction, who were in a weight loss program, and/or those with any comorbidity (except obesity) were not included. Review articles, experimental studies, guidelines, theses, dissertations and abstracts, as well as studies that did not meet the aforementioned inclusion criteria, were also excluded.

### Data analysis and extraction

He extracted data were organized in a Microsoft Excel (Microsoft Corp., Washington, United States) spreadsheet and included: title, authors, place of performance, year of publication, study design, objective, characteristics of the individuals, sample size, measurements of body fat percentage, inflammatory markers and main results (Table 1). The primary outcome was the presence of inflammatory markers associated with body fat percentage in adolescents. Data are presented in ascending order of year of publication.

### Meta-analysis

Articles that presented correlation coefficients between body fat percentage and inflammatory markers were included in the meta-analysis. The generic inverse variance clustering method was used to combine correlations from different studies into a clustered correlation estimate (21). Correlations were systematized and meta-analysis was performed using RStudio software (Posit software, PBC, Boston, MA, United States) version 4.0.4 with the metabin function included in the meta package. with the metabin function included in the meta package. In addition, the information was plotted on a florestplot (22). To detect studies that contributed to heterogeneity, the effect graph of each study was evaluated with the Influence Analysis function of the meta package (23). The heterogeneity between the studies was quantified by means of proportion (by the *I*^2^ statistic), in which values above 25, 50, and 75% are considered low, moderate and high heterogeneity, respectively. The level of statistical significance established was *p* < 0.05.

### Risk of bias assessment

The risk of bias was independently assessed by the critical assessment tool recommended by the Joanna Briggs Institute, by two researchers, with the aid of the checklist (Supplementary material—Appendix II) for cross-sectional studies, composed of 08 questions (24), and for cohort studies, consisting of 11 questions, which can be answered as: yes, no, unclear or not applicable. “Yes” answers indicate a low risk of bias, and “no” answers are expected to be high risk of bias.

### Review quality assessment

The quality of this systematic review was evaluated according to the Assessment of Multiple Systematic Reviews, AmStar, method. This approach considers several key aspects, including the definition of the central question of the review, the inclusion criteria, and peer assessment of researchers. Procedures for consensus decision in case of disagreements in the inclusion or exclusion of articles, and broad search considering different bases and gray literature sources. Additionally, characterization and quality assessment of included studies, methods used to combine study findings, probability of bias in published results, and possible conflicts of interest (25).

## Results

Figure 1 presents the detailed flowchart of the selection of studies, according to the PRISMA protocol. The search identified 7,592 articles, and after selection, 31 studies were included, representing 29 cross-sectional and two prospective cohorts.

### Description of studies

The articles included in the review were published from 1998 to 2022 and carried out in different locations: Brazil (9, 11, 20, 26–31); EUA (30–34); Taiwan (35–38); India (39); Spain (40, 41); Iran (42, 43); Greece (44); Poland (45, 46); South Afica (47, 48); Denmark (49); Finland (50); Iceland (51); and Portugal (52). The sample size ranged from 27 (32) a 3,877 (28) and the age of the adolescents evaluated was from 10 to 19 years old, comprising the entire age group age group that involves adolescence (53). Individuals of both sexes were evaluated in most studies: seven female (9, 27, 29, 37, 43, 44, 46, 50) and one male (38) (Table 1).

**TABLE 1:** Characteristics of the selected studies.

References	Country	Population/ age (years)	Study design	Objective	BF assessment method	%BF Mean (SD) Median (Min-Max)	Inflammatory markers Mean (SD) Median (Min-Max)	Main results
Arslanian et al. (32)	U. S.	27 adolescents (13M; 14F) Age: 13.5 ± 0.2	Cross-sectional	To evaluate the relationships of leptin with body composition, puberty and insulin sensitivity in healthy prepubertal children and pubertal adolescents.	Bioimpedance	15.1 ± 1.8	Leptin (ng/mL) 10.9 ± 2.2	Leptin correlated with %BF (*r* = 0.63). In adolescents, leptin reflects body fat stores.
Brandão et al. (26)	Brazil	175 adolescents (95 M; 80 F) Age: 10–18	Cross-sectional	To study the physiological changes of leptin during puberty in both sexes and its relationship with body composition and sexual maturation.	DEXA	M: 20.00 ± 8.03 F: 28.66 ± 6.86	Leptin (ng/mL) M: 3.63 ± 4.26; F: 8.53 ± 7.67	Leptin concentration correlated with %BF positively in both sexes (F: *r* = 0.76; M: *r* = 0.85).
Huang et al. (35)	Taiwan	232 adolescents (125 M; 107 F) Age: 15.7 ± 1.8 M; 15.4 ± 1.9 F	Cross-sectional	To examine the relationships between plasma adiponectin, anthropometric indices and various metabolic factors among healthy non-diabetic adolescents.	DEXA	M: 32.5 ± 8.4 F: 39.7 ± 6.0	Adiponectin (μg/mL) 22.87 ± 11.41 (M); 30.79 ± 14.48 (F)	Adiponectin was negatively correlated with %BF (*r* = −0.227).
Huang et al. (36)	Taiwan	402 adolescents (162 M; 240 F) Age: 15.7 ± 1.9 M; 15.8 ± 1.9 F	Cross-sectional	To investigate the association between plasma leptin levels and the IR index, with the various anthropometric indices, body fat mass and lipids in non-diabetic adolescents.	DEXA	M: 33.2 (28.5–38.3) F: 40.0 (35.1–44.0)	Leptin (ng/mL) M: 7.43 (3.62–12.52); F: 15.50 (9.84–23.34)	Leptin levels were higher in females, and for both sexes, leptin correlated with %BF (*r* = 0.815).
Vikram et al. (39)	India	62 male adolescents Age: 15.9 ± 1.2	Cross-sectional	To evaluate the relationship of anthropometry, body fat percentage (BF%), lipoproteins, fasting insulin, insulin resistance, and ultrasensitive CRP (us-CRP) levels, with serum adiponectin levels in postpubertal Asian Indian men.	Bioimpedance	36.0 ± 5.8	Adiponectin (μg/mL): 10.3 ± 4.1 hs-CRP (mg/dL): 3.94 ± 2.83	Both markers correlated with %BF; Adiponectin (μg/mL) – *r* = −0.24 CRP (mg/dL) – *r* = 0.42
Warnberg et al. (40)	Spain	472 adolescents (248M; 224F) Age: 15.28 ± 1.19	Cross-sectional	To establish the possible relationships of serum inflammatory proteins with estimates of body fat and body fat distribution in an apparently healthy adolescent population.	DEXA	M: 15.16 ± 5.29 F: 23.61 ± 4.35	CRP (mg/L): M: 1.17 ± 1.62; F: 0,83 ± 0,86 IL-6 (pg/L): M: 3,230 ± 1,954; F: 3,526 ± 2,196 TNF-a (pg/L): M: 2,290 ± 1,624; F: 2,275 ± 2,451	Of the analyzed markers, only CRP (mg/L) was significantly correlated with %BF, in both sexes (M: *r* = 0.264; F: *r* = 0.281).
Kelishadi et al. (42)	Iran	512 adolescents (254 M; 258 M) Age: 10–18	Cross-sectional	To determine the association of serum C-reactive protein with generalized and abdominal obesity, body fat composition, metabolic syndrome and oxidative stress markers in young people.	Bioimpedance	M: 15.4 ± 2.3 (10–13) 19.7 ± 3.6 (14–18) F: 21.8 ± 3.9 (10–13) 24.8 ± 4.2 (14–1)	PCR (mg/L) M: 1.2 ± 0.1 (10–13); 1.0 ± 0.1 (14–18 years old) F: 1.0 ± 0.2 (10–13); 1.1 ± 0.3 (14–18 years old)	CRP (mg/L) showed a positive correlation (*r* = 0.12) with %BF, for both sexes.
Stylianou et al. (44)	Greece	55 adolescents (26M; 29 F) Age: 12.8 ± 1.8	Cross-sectional	Investigate any correlation between ghrelin and leptin and body fat percentage.	Bioimpedance	34.33 ± 5.14	Leptin (ng/dL) 34.74 ± 17.93	Leptin was positively correlated with %BF (*r* = 0.617), regardless of BMI.
Blogowska et al. (45)	Poland	65 female children and adolescents, in premenarche Age: 7.9–13.5	Prospective cohort	To assess changes in body composition in girls during the premenarcheal stages of development, including the early stage without clinical manifestations of changes in primary, secondary and tertiary sexual characteristics.	Infrared Interact (Futrex)	23.9 ± 6.8	Leptin (μg/L): 11.3 ± 7.2	Leptin showed a positive correlation with %BF in the postmenarcheal maturation stage (*r* = 0.79)
Caballero et al. (30)	U. S.	38 children and adolescents (M:19; F:19) Age: 10–18	Cross-sectional	To evaluate plasma markers of endothelial dysfunction, vascular inflammation, and procoagulation in obese Hispanic/Latino children and adolescents with normal glucose tolerance and to determine their relationship with body composition and glucose indices and lipid metabolism.	DEXA	E: 24.0 ± 6.0 O; 42.0 ± 9.0	TNF-α (pg/mL) IL-6 (pg/L) hs-CRP (mg/L) Adiponectin (ng/mL) [NA]	Adiponectin levels were lower in the obese group than in the eutrophic group. A positive correlation was observed between %BF and hs-CRP (*r* = 0.70). The other markers showed no correlation.
McVean et al. (31)	U. S.	75 adolescents (31M; 44 F) Age: 12.5 ± 0.6	Cross-sectional	To analyze the relationship between CRP, adiponectin, body composition and physical fitness levels in non-obese children.	DEXA	24.5 ± 0.6	CRP (mg/dL): 0.032 ± 0.04 Adiponectin (ng/mL): 16.60 ± 6.52	CRP showed the strongest correlation with body fat. And %BF remained a significant predictor of CRP in the multiple regression analysis (*p* < 0.001), adjusted for age, sex, and cardiovascular fitness. CRP correlated with %BF (*r* = 0.46) Adiponectin correlated with % BF (*r* = −0.04, NS)
Schoppen et al. (41)	Spain	833 Adolescents (397M; 436F) Age: 12–16	Cross-sectional	To describe the serum concentrations of leptin and adiponectin in a sample of pubescent Spanish adolescents and to assess their association with anthropometric parameters and body composition.	Bioimpedance	[NA]	Leptin (ng/mL): M: 6.1 ± 8.1; F: 16.0 ± 10.0 Adiponectin (ng/mL): M: 11.3 ± 6.7; F: 15.4 ± 8.0	Leptin was correlated with %BF, in both sexes (F: *r* = 0.650; M: *r* = 0.828). For adiponectin, the correlation was not significant. Leptin concentrations were higher for obese and overweight adolescents compared to normal weight.
Karen et al. (33)	U. S.	166 adolescents (78M; 88F) Age: 14–19	Cross-sectional	To determine sex differences in inflammatory markers and the association between adiposity and inflammation in a sample of African-American adolescents.	DEXA	M: 12812.55 ± 8630.3 g F: 22831.88 ± 11773.2 g	Leptin (ng/mL); M: 4.80 ± 6.6; F: 22.24 ± 19.3 Adiponectin (μg/mL); M: 5.41 ± 3.2; F: 6.99 ± 4.3 IL-6 (pg/mL); M: 1.29 ± 1.3; F: 1.80 ± 1.7 CRP (μg/mL); M: 1.01 ± 1.8; F: 1.27 ± 1.8	The %BF correlated with the markers as follows: - Male gender: positive correlation with CRP (*r* = 0.252), leptin (*r* = 0.588) and IL-6 (*r* = 0.211); negative with adiponectin (*r* = −0.262). – Female gender: positive correlation with CRP (*r* = 0.486), leptin (*r* = 0.475) and IL-6 (*r* = 0.227); negative with adiponectin (*r* = −0.178)
Kruger; Pretorius; Schutte (47)	South Africa	184 adolescents (69 M; 109 F) Age: 15.9 ± 1.4 M; 15.5 ± 1,33 F	Cross-sectional	To compare the inflammatory status of children with differences in nutritional status.	Air displacement plethysmography	M: 18.3 ± 6.80 F: 28.7 ± 6.50	TNF-α (pg/mL): M: 0.8 (0.2–1.6); F: 1.1 (0.05–2.1) IL-6 (pg/mL): M: 2.50 (1.0–4.6); F: 1.90 (1.1–3.6) CRP (mg/L): M: 0.36 (0.2–1.1); F: 0.34 (0.2–1.0)	Markers did not correlate with %BF.
Serrano et al. (9)	Brazil	113 female adolescentes Age: 14–18	Cross-sectional	To assess body composition, anthropometric, biochemical and clinical changes in female adolescents.	Bioimpedance	28.72 ± 5.14	Leptin (ng/mL): 14.4 ± 15.37 CRP (mg/dL): 3.572 ± 3.732	Only leptin (*r* = 0.193) correlated with %BF. CRP showed no significant correlation (*r* = 0.112, NS)
Zeelie; Moss; Kruger (48)	South Africa	232 adolescents (99M; 133F) Age: 15–19	Cross-sectional	To determine the relationship between body composition and selected markers of metabolic syndrome in black adolescents.	Air displacement plethysmography	M (%BF ≤ 20%): 14.3 ± 2.8; (%GC > 20%): 26.0 ± 5.0 F (%BF ≤ 25%): 20.3 ± 4.6; (%BF > 25%): 31.8 ± 4.8	Leptin (ng/mL) M (%GC ≤ 20%): 2.38 ± 0.8; (%GC > 20%): 6.5 ± 5,0 F (%BF ≤ 25%): 10.6 ± 6.6; (%GC > 25%): 21.9 ± 11.9	Leptin (ng/mL) was higher in adolescents with higher BF%, and was positively correlated with BF% (*r* = 0.66).
Bundy et al. (34)	U. S.	441 adolescents (M: 222; F: 219) Age: 14–18	Cross-sectional	To determine gender or race differences in the associations between adiposity and leptin, and whether adiponectin moderates these relationships.	DEXA	White M: 19.6 (8.6) F: 29.4 (6.9) Black M: 16.9 (8.4) F: 30.0 (8.4)	Adiponectin (μg/mL) White M: 15.7 (7.9); F: 21.7 (9.8) Black M: 14.9 (10.0); F: 15.6 (8.5) Leptin (ng/mL) White M: 2.8 (0.3–61.2); F: 12.1 (1.3–57,2) Black M: 2.3 (0.2–37.0); F: 17.3 (2.8–77.3)	%BF was associated with leptin (*R*^2^ = 0.36), controlled by race and gender. Furthermore, lower adiponectin was associated with higher leptin (*R*^2^ = 0.38).
Plonka et al. (46)	Poland	59 female adolescents Age: 12.5 ± 1.67	Cross-sectional	To evaluate the correlation between physical activity and serum leptin concentration in the blood in relation to body mass composition and to select fat indexes of the examined girls.	Bioimpedance	22.97 ± 9.72	Leptin (ng/mL); 12.34 ± 7.33	Leptin was positively correlated with %BF (R^2^: 0.657).
Bugge et al. (49)	Denmark	413 adolescents Age: 13.4 ± 0.34 M; 13.3 ± 0.33F.	Cross-sectional	To assess associations between inflammatory markers and cardiovascular disease (CVD) risk factor clusters and examine how inflammatory markers and CVD risk are related to fat and cardiorespiratory fitness in adolescents.	Sum of 4 SF	M: 32.0 ± 17.4 F: 38.3 ± 15.3	Adiponectin (μg/mL); M: 12.0 ± 7.5 F: 12.7 ± 8.0 IL-6 (pg/mL); M: 0.57 ± 0.45 F: 0.72 ± 1.18 TNF-α (pg/mL); M: 0.64 ± 0.52 F: 0.59 ± 0.55 CRP (mg/L); M: 0.66 ± 1.1 F: 0.6 ± 0.8	The sum of the 4 SF was negatively correlated with adiponectin (*r* = –0.145) and positively associated with CRP (*r* = 0.247), IL-6 (*r* = 0.151) and TNF-α (*r* = 0.101). There were no significant correlations for the group below the median of the sum of the 4 SF, for the group above the median, there was a negative correlation with adiponectin (*r* = –0.139) and a positive correlation with (*r* = 0.296).
Mirhosseini et al. (43)	Iran	477 female adolescents Age: 16.4 ± 0.9	Cross-sectional	To assess the association between measures of adiposity and established cardiovascular risk factors in adolescent girls.	Bioimpedance	21.3 ± 7.1	CRP (mg/L): 1.3 ± 2.6	CRP was significantly associated with %BF (*r* = 0.10).
Wen et al. (50)	Finland	396 female adolescents Age: 11.2 ± 0.8	Prospective cohort	To assess the developmental trajectories of levels of fat mass and high-sensitivity C-reactive protein (hs-CRP) and factors that could explain the relationship between fat mass and hs-CRP in prepubertal to early adulthood girls.	DEXA	[NA]	Leptin (ng/mL): 19.5	Regression analysis showed that Leptin (ng/mL) was associated with %BF (β = 1,121)
Coutinho et al. (27)	Brazil	53 female adolescents Age: 13–17	Cross-sectional	To investigate the association between total and abdominal adiposity with metabolic parameters and inflammatory markers in female adolescents.	DEXA	39.8 ± 7.1	Adiponectin (ng/mL): 5.8 (4.9–9.3)^‡^ Leptin (pg/mL): 40.4 (25.9–59.5)^‡^ TNF-α (pg/mL): 1.7 (1.2–2.4)^‡^ CRP (ng/mL): 1.4 (0.5–4.7)^‡^ IL-6 (pg/mL): 1.2 (0.9–2.0)^‡^	There was a significant positive correlation between %BF with CRP (ng/mL) (*r* = 0.20) and leptin (*r* = 0.58). For the other markers there was no significant correlation [adiponectin (*r* = 0.02), TNF-α (*r* = 0.04), IL-6 (*r* = 0.02)].
Hinriksdóttir et al. (51)	Iceland	245 adolescents (129 M; 116 F) Age: 18	Cross-sectional	To assess the relationship between adiposity, different expressions of fitness and CRP in late adolescence using direct measures of fitness and fat.	DEXA	M: 20.4 ± 7.7 F: 31.0 ± 5.8	CRP (mg/L): M: 1.0 ± 1.2; F: 1.4 ± 1.7	CRP (mg/L) was positively correlated with %BF, in both sexes (*r* = 0.33).
Wu et al. (37)	Taiwan	500 adolescents (228 M; 272 F) Age: 14.16 ± 0.79 M; 14.30 ± 0.74 F	Cross-sectional	To investigate risk factors associated with cardiovascular diseases and their relationship with BMI, body fat mass and plasma leptin level among adolescents in Taitung, Taiwan.	Bioimpedance	M: 18.75 ± 6.70 F: 24.28 ± 6.99	Leptin (ng/mL): M: 5.52 ± 9.94; F: 9.91; 9.89	Leptin was positively correlated with %BF, in both sexes (*r* = 0.59).
Menezes et al. (28)	Brazil	3,877 adolescents (1,938M; 1,939F) Age: 18	Cross-sectional	Cross-sectional and prospective assessment of the association of different measures of obesity (BMI, WC and %BF) and IL-6, CRP and adiponectin at 22 years of age, in a population-based Birth Cohort in Southern Brazil.	Air displacement plethysmography	M: 16.7 ± 8,9 F: 32.7 ± 7.8	IL-6 (pg/mL): 1,61 ± 1.95 (M); 1.78 ± 1.95 (F) CRP (mg/L): 1.52 ± 2.93 (M); 3.15 ± 4.72 (F) Adiponectin (μg/mL): 14.26 ± 7.04 (M); 17.04 ± 7.91 (F)	Regression analysis showed that IL-6 (pg/mL), CRP (mg/L) and Adiponectin (μg/mL) had a highly significant direct association with %BF (*p* < 0.001), in both sexes, even after adjustments.
Miranda et al. (29)	Brazil	405 female adolescents Age: 15.9 ± 1.27	Cross-sectional	To evaluate the association between lifestyle classes and body composition groups with risk factors for cardiometabolic diseases and pro- and anti-inflammatory biomarkers in female adolescents.	DEXA	29.0 (26.2–34.5)	IL-6 (pg/mL): 1.95 (1,27–2,87) TNF-α (pg/mL): 2.05 (1.24–2,8) Leptin (pg/mL): 4841.4 (2818.2–7858.7) IL-10 (pg/mL): 1.38 (1.0-2.07)	Regression analysis showed that leptin was positively associated with %BF. Leptin (pg/mL) - *R*^2^: 0.34 IL-6, TNF-α and IL-10 did not associate with%BF.
Bragança et al. (11)	Brazil	403 adolescents (269M; 134F) Age: 18–19	Cross-sectional	To compare biomarkers in groups of adolescents classified simultaneously by BMI and % BF.	Air displacement plethysmography	Division into 4 groups of NS: G1- Eutrophic G2- Obese of normal weight G3- Excess weight and adequate % BF G4- Excess weight and high % GC	IL-6 (pg/mL) G1: 1.5 (1.0–2,7), G2: 2.4 (1.3–3.5), G3: 1.5 (1.1–4.1), G4: 2.7 (1.5–5.3) TNF-α (pg/mL) G1: 5.5 (3.9–8.1), G2: 5.5 (3.7–7.5), G3: 6.5 (3.9–8.5), G4: 5.5 (4.0–8.1) CRP (ng/mL) G1: 0.06 (0.02–0.14), G2: 0.10 (0.03–0.15), G3: 0.14 (0.05–0.19), G4: 0.14 (0.08–0.69)	G4 had a higher average for IL-6 and CRP compared to G1 and G3. IL-6 and CRP levels were significantly higher in adolescents with higher %BF.
Agostinis-Sobrinho et al. (52)	Portugal	529 adolescents (262M; 267F) Age: 12–18 Age: 14.3 ± 1.7 M; 14.2 ± 1.7 F	Cross-sectional	To examine differences in insulin resistance (IR) between adiposity levels and to verify whether high adiponectin levels attenuate the detrimental relationship between adiposity and IR in adolescents.	Bioimpedância	20.6 ± 8.3 F: 25.3 ± 7.0 M: 15.8 ± 6.6	Adiponectin (mg/mL) 11.6 ± 5.4 F: 12.9 ± 5.7 M: 10.2 ± 4.8	Adolescents in the 4th hurt of adiponectin had 0.77 SD for %BF compared to the 1st quartile. Adiponectin levels were significantly higher in adolescents with excess body fat.
Sodré et al. (20)	Brazil	74 adolescents (32M; 42F) Age: 10–19	Cross-sectional	To verify the possible relationship between serum uric acid concentrations and insulin resistance in adolescents.	Bioimpedância	25.97 ± 10.47	UA (mg/dL): 5.28	%BF was higher for the group with hyperuricemia (29.48 ± 8.96) compared to the others (24.05 ± 9.14).
Kuo et al. (38)	Taiwan	387 adolescents (218 M; 169 F) Age: 17.4 ± 1.3	Cross-sectional	To explore the relationship between anthropometric indices and hyperuricemia among adolescent athletes.	Bioimpedância	M: 12.6 ± 4.7 F: 24.2 ± 6.1	UA (mg/dL) M: 6.4 ± 1.1 F: 4.9 ± 1.1	The chance of having hyperuricemia in individuals with %BF in Quantile 1 and Quantile 5 was higher than in the three middle quantile, in both sexes: - M: 1st Quantile- OR: 2.16 (1.00–4.68); 5th Quantile-OR: 2.21 (1.05–4.66) - F: 1st Quantile- OR: 2.92 (1.08–7.91); 5th Quantile-OR: 8.90 (3.51–22.6)
Zhang et al. (54)	China	1,021 adolescents (497 M; 524 F) Age: 16.0 ± 1.8 M; 16.4 ± 1.9 F	Cross-sectional	To investigate gender-specific patterns of plasma leptin during adolescence, assess which measures of adiposity are most strongly associated with plasma leptin, and estimate the extent to which leptin-adiposity associations are influenced by genetic factors.	DEXA	M: 11.8 ± 5.3 F: 27.2 ± 5.7	Leptin (ng/mL) M: 1.99 ± 2.13 F: 8.20 ± 5.48	The higher the %BF quarts, the higher leptin values. Association between %BF and leptin: M: *R*^2^ = 0.397 F: *R*^2^ = 0.214

%BF, Body fat percentage; WC, Waist circumference; CVD, Cardiovascular disease; F, Female; BF, Body fat; IL-6, Interleukin-6; BMI, Body Mass Index; M, Male; CRP, C-reactive protein; hs-CRP, Ultrasensitive C-reactive protein; IR, Insulin resistance; SF, Skinfold; DEXA, Dual Energy X-ray Absorptiometry; SD, Standard Deviation; E, Eutrophic; IL-6, Interleukin-6; NA, Data not shown; NS, Not significant data; O, Obese; TNF-α, Tumor Necrosis Factor-α. OR, Odds Ratio; R^2^, Coefficient of Determination; r, Correlation Coefficient; β, Linear Regression Coefficient.

### Main results

The studies used different techniques to assess the body fat percentage (%BF): bioimpedance (9, 20, 32, 38, 39, 41–44, 46, 52); dual energy X-ray absorptiometry (DEXA) (20, 26, 27, 29–31, 33–36, 40, 50, 51, 54); air displacement plethysmography (11, 28, 47, 48); sum of the four skinfolds (49); and infrared interactance (Futrex) (45). Each of these methods has advantages and disadvantages, with DEXA and air displacement plethysmography being considered the gold standard, however, they are expensive methods that are not widely available. Bioimpedance, despite being widely used for its practicality, speed, and for being non-invasive, varies according to the individual’s hydration, which can lead to under- or overestimation of the body fat value. Infrared ray interactance is fast, safe, and non-invasive, however, it is less specific than other methods, especially in cases of excess adiposity. The sum of the four folds is a simple, low cost method, however, it requires a highly trained examiner.

The inflammatory markers evaluated were: CRP (9, 11, 27, 28, 30, 31, 33, 39, 40, 42, 43, 47, 49, 51); leptin (9, 26, 27, 29, 32–34, 36, 37, 41, 44–46, 48, 50, 54); adiponectin (28, 30, 31, 33–35, 39, 41, 52); tumor necrosis factor-α (TNF-α) (11, 27, 29, 30, 40, 47, 49); interleukin-6 (IL-6) (11, 27–30, 33, 40, 47, 49); interleukin-10 (IL-10) (29), considered mediators of inflammation, secreted by adipose tissue or by other organs, through the stimulation of pro-inflammatory cytokines secreted by adipocytes, such as CRP, synthesized by hepatocytes by stimulation of IL-6 and TNF-α. Also, uric acid was evaluated (20, 38) which, although not a marker of inflammation, at high levels, such as above 5 mg/dL, is involved in metabolic complications associated with adipose tissue.

Despite the various markers described in the researched literature, only CRP (27, 30, 31, 33, 39, 40, 42, 43, 51); leptin (9, 26, 27, 32–34, 36, 41, 44–46, 48); IL-6 (33); and adiponectin (31, 33, 35, 39) were correlated with %BF in the adolescentes of the studies included in this review (Table 1).

**FIGURE 2:**
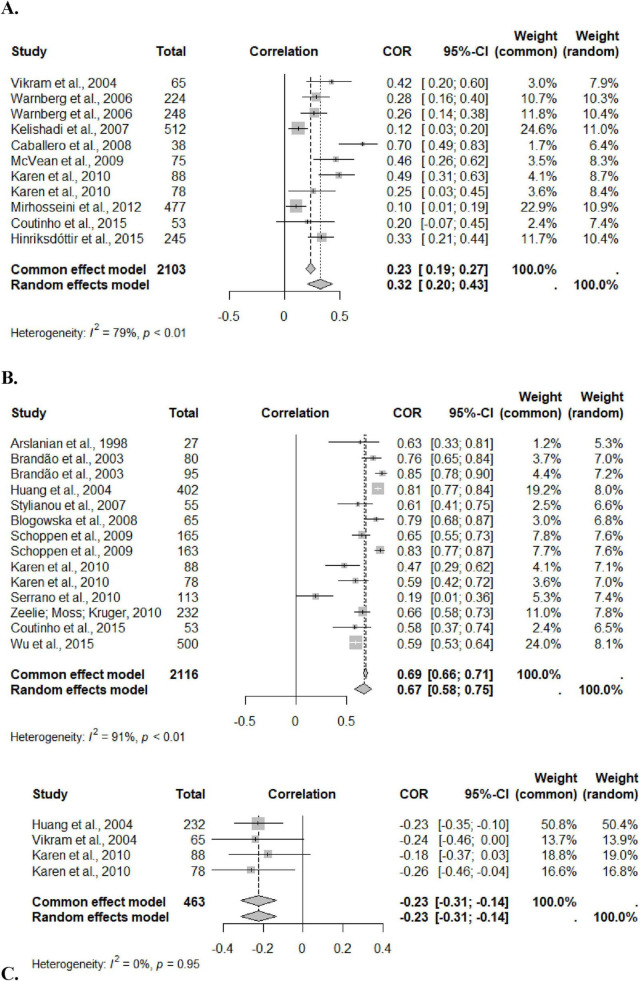
Forestplot for correlation coefficients between body fat percentage and Leptin **(A)**, CRP **(B)** and Adiponectin **(C)** in adolescents. This figure represents the individual effect of each study and the size indicates the sample weight; The black line represents the confidence interval (CI) of each study; The diamond systematizes the correlation between the inflammatory marker and the percentage of fat from all studies and the horizontal edges indicate the CI.

The meta-analysis included 4,682 adolescents, aged 10–19 years, of both sexes. The systematization included three markers: CRP, leptin and adiponectin. Analyes were performed by sex, but there was no significant result due to the low number of studies that had correlation data separated by sex, showing that it was not a mediating factor. The correlation between %BF and leptin was 0.67 (95%CI: 0.58; 0.75), heterogeneity of 91%, and with CRP it was 0.32 (95%CI: 0.20; 0.43), heterogeneity of 79%. The result of the randomized model was considered, due to the high heterogeneity presented in the two correlations. For adiponectin, the correlation was −0.23 (95%CI: −0.31; −0.14), heterogeneity of 0%, considering the result of the fixed effect, given its low heterogeneity (*I*^2^ = 0) (Figure 2).

Despite the high heterogeneity for the first two analyses, all studies included in the meta-analysis showed a positive correlation, corroborating the systematized result. Examining the influence graph, it is evident that the exclusion of any study is capable of significantly reducing heterogeneity (Supplementary material—Appendix III).

In addition, it was considered in the meta-analysis whether the methods of %BF assessment influenced the outcomes of inflammation. Significant results were found only for leptin and CRP. The correlation coefficient between %BF and leptin and between %BF and CRP were higher when the method used to assess fat was densitometry, compared to bioimpedance, with high heterogeneity in both analyses (Supplementary material—Appendix IV).

Finally, systematization of the correlation between BMI and the variables leptin, CRP and adiponectin was also performed, and it was observed that the correlation between BMI and CRP was 0.30 (95%CI: 0.24; 0.36), heterogeneity of 28%; with leptin it was 0.56 (95%CI; 0.46; 0.64), heterogeneity of 76%; and with adiponectin it was −0.20 (95% CI; −0.27; −0.13), 0% heterogeneity. These results confirm the superiority of the percentage of body fat to predict the responses of inflammatory markers, when compared to BMI, indicating the importance of its evaluation in clinical practice (Supplementary material—Appendix V).

### Risk of bias assessment

**FIGURE 3:**
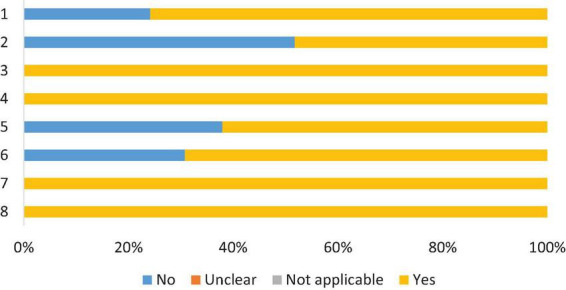
Risk of bias assessment, according to the Joanna Briggs Institute critical assessment checklist, for cross-sectional studies. 1. Criteria for inclusion in the sample cleary defined; 2. Study subjects and the setting described in detail; 3. Exposure measured in a valid and reliable way; 4. Objective and standard criteria for measurement; 5. Confounding factors identified; 6. Strategies to deal with confounding factors; 7. Outcomes measured in a valid and reliable way; 8. Appropriate statically analysis.

**FIGURE 4:**
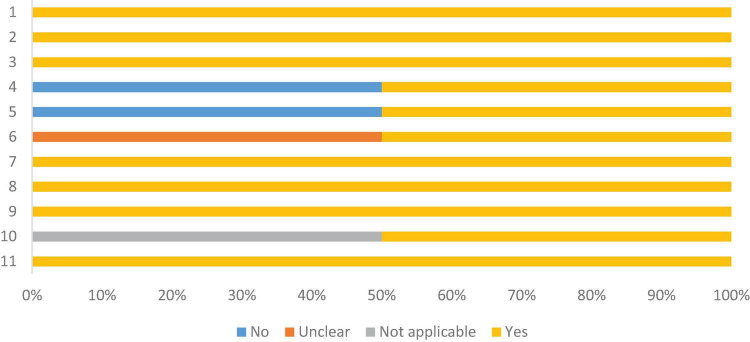
Risk of bias assessment, according to the Joanna Briggs Institute critical assessment checklist, for cohort studies. 1. Similar groups recruited from the same population; 2. Exposures in a similar way to designate as persons to exposed and unexposed groups; 3. Validly and reliably measured exposure; 4. Identification factors; 5 Strategies for dealing with confounders; 6. Groups were free/participating at baseline (or at time of exposure); 7. Intermediate results validly and reliably; 8. Follow-up time was observed and sufficient for results to occur; 9. Follow-up was complete and, if not, reasons for loss of follow-up were described and exploratory; 10. Strategies were used to address incomplete follow-up; 11. Appropriate statistics.

Most studies were of the cross-sectional type, with two of the prospective cohort type (45, 50). Therefore, the risk of bias was assessed using two tools: one for cross-sectional and the other for cohort. All had validly and reliably measured exposure and results, valid objective and measurement criteria, and appropriate statistical analysis. Sixteen (51.6%) did not describe in detail the individuals and the evaluated environment, and twelve (38.7%) did not identify confounding factors. Despite this, the articles included in this review showed 82.3% of the answers as “yes” in the critical assessment recommended by the Joanna Briggs Institute for cross-sectional studies. And 81.8% for cohort studies, as shown in Figures 3, 4, respectively, which indicates a low risk of bias, according to Moola et al. (24) (Supplementary material—Appendix II).

### Review quality assessment

All criteria proposed by the method used for quality assessment were met in this review (25) (Supplementary material—Appendix VI).

## Discussion

This review presents a systematization of published articles on inflammation and body fat composition in adolescents, and evidences a strong correlation of the inflammatory markers CRP, leptin and adiponectin with the body fat percentage in adolescents, portrayed by the result of the meta-analysis. Despite the high heterogeneity of the studies, justified by the inclusion of different ethnic populations, as well as by different techniques for assessing body fat, we can consider this result important to describe the relationship between the inflammatory profile of adolescents and adiposity.

The main results of the studies included (9, 11, 20, 26–52, 54) in this review showed that the body fat percentage is positively associated with the presence of inflammatory markers leptin, adiponectin, CRP and IL-6 in adolescents of both sexes, and that the higher the percentage of fat, the higher the level of these markers. Furthermore, the chance of presenting hyperuricemia is greater in adolescents with a high percentage of body fat.

Obesity, resulting from positive energy balance, is characterized by increased white adipose tissue, associated with hypertrophy or even hyperplasia of white adipocytes. This abnormal increase is associated with systemic metabolic changes such as hyperglycemia, insulin resistance and dyslipidemias. In addition, white adipose tissue is currently considered a metabolically active organ, of endocrine nature, responsible for the expression and/or secretion of adipokines and other inflammatory proteins, which act locally or systemically, thus characterizing the inflammation present in excess adiposity (16).

In the initial periods of excess caloric intake, the organism performs a physiological expansion of white adipose tissue with the secretion of acute proinflammatory mediators that will modulate triglyceride deposits in order to avoid ectopic fat accumulation. This process is necessary and physiologically adaptive. However, when a prolonged positive energy balance occurs, obesity progresses and this process is suppressed, resulting in metabolic stress, with a low-grade chronic inflammatory state, with various pathological consequences (55, 56).

Although it is not known exactly what the primary stimulus for inflammation associated adipose tissue is, it is known that there are several metabolic processes involved in hypertrophy and hyperplasia in excess of adipose cells resulting from excessive caloric intake. Among these processes, there is fibrosis and mechanical stress caused by the rapid expansion of adipocytes. Also, there are: hypoxia resulting from insufficient vascularization of the expanding white adipose tissue, the death of adipocytes due to their inability to store the fat and glucose overload, and the infiltration of macrophages, which account for 60% of obesity adipose tissue cells. As well as the high levels of lipopolysaccharides in the circulation derived from the intestinal microbiota, due to the excess of lipid absorption in the enterocytes and the compromise of the intestinal barrier (55, 57, 58).

The expansion of adipose tissue causes inflammatory changes that lead to low-grade systemic inflammation, with increased levels of cytokines. The accumulation of inflammatory cells occurs more due to the accumulation of visceral fat than of subcutaneous fat. This inflammation differs from that normally used against invading hosts of the organism because it is chronic, low-grade, and affects the metabolism of nutrients in adipose, hepatic, muscular and pancreatic tissue. This is because it promotes insulin resistance, which is a link between obesity and inflammation (16).

The studies presented here showed that the inflammatory markers correlated with a high body fat percentage are IL-6, leptin, CRP and adiponectin. IL-6 is secreted by adipocytes due to excessive lipolysis and an increase in free fatty acids, so that excess visceral fat increases the levels of this cytokine by 2–3 times (59). It is involved in the processes of carbohydrate and lipid metabolism, leading to hyperinsulinemia, by altering the expression of insulin receptors, and increasing levels of fatty acids and glycerol, by inhibiting lipoprotein lipase and increasing lipolysis (60–62). In adolescentes with obesity, high concentrations of IL-6 have been a reflection of a proinflammatory and prothrombotic state (63), being strongly associated with the presence of insulin resistance (64). Even in eutrophic adolescents, but with a high %BF, IL-6 was correlated with other inflammatory cytokines, such as TNF-α and with the development of insulin resistance (8) as well as, with modulating the synthesis of CRP, showing the role of adipose tissue in inflammation.

Leptin, an important adipokine, acts on molecular pathways such as PI3K/AKT and mTOR is encoded by the obesity gene and is involved in the process of regulating food intake. When fat tissue, especially visceral fat, is in excess, there is also an excess of these cytokines and, in response, the body, via the central nervous system, promotes resistance to their action, exacerbating obesity and leading to hyperglycemia and insulin resistance (65).

In adolescents, leptin has shown a strong correlation with all body fat parameters, mediating the relationship between body adiposity and blood pressure elevation in adolescents of both sexes (66). In females, there is a greater number of significant correlations between leptin and cardiometabolic risk factors (67).

Another important adipokine is adiponectin, which has an anti-inflammatory role in tissues, and acts as an insulin sensitizer. It has reduced levels in obesity, which compromises the activation of AMP kinase and worsens metabolic dysfunction, promoting a insulin resistance, liver fibrosis and, consequently, inflammation (68). Adiponectin in adolescents was correlated with obesity, insulin resistance and hyperlipidemia (69), as well as the presence of metabolic syndrome in these individuals (70).

C-reactive protein (CRP) is an inflammation marker synthesized by hepatocytes, which also has its production regulated by other cytokines, such as TNF-α and IL-6, produced by adipocytes. It is an acute phase protein involved in system activation, complement and phagocytic cell recruitment (71, 72). In adolescents, CRP was positively correlated with measures of body and visceral adiposity (63), and also with insulin resistance in adipose tissue (64, 73). This suggests that the increase in CRP occurs due to the inflammatory state of obesity, and may be an important indicator in the diagnosis of cardiovascular and metabolic complications in adolescent (74).

With regard to uric acid, the end product of purine metabolism, it is associated with chronic inflammation of adipose tissue (75). Its evaluation is important because, when elevated, at levels above 5 mg/dL, it is associated with cardiometabolic complications from an early age (76). In adolescents, insulin, fasting glucose, blood pressure and %BF values have been significantly higher in those with hyperuricemia (75, 76).

Epigenetic alterations in genes associated with the inflammatory markers discussed, such as leptin, C-reactive protein, and adiponectin, play a crucial role in pediatric obesity. These alterations, including DNA methylation and histone modifications, modulate the expression of inflammatory cytokines, contributing to a sustained inflammatory state and perpetuating an adverse metabolic profile. Such mechanisms are closely linked to the activation of the NF-κB pathway, a key regulator of pro-inflammatory gene expression in adipose tissue and other target organs. This intricate interplay between epigenetic changes and inflammatory pathways highlights the molecular basis of the elevated cardiometabolic risk observed in adolescents with a higher body fat percentage, as demonstrated in the present study (77).

It is understood, therefore, that the dysfunction of adipose tissue with an increase in the inflammatory process is the central mechanism for the development of the many metabolic complications resulting from obesity, since it is a tissue that communicates with many other organs, releasing inflammatory molecules that affect the homeostasis of the whole organism (76). Thus, the analysis of the body fat percentage is necessary to understand the inflammatory profile of adolescents, since the use of BMI in the assessment of nutritional status does not allow the correct identification of fat mass, thus underestimating the prevalence of obesity (12–14). This is so, since more than a quarter of adolescents with a high %BF are classified as eutrophic when using only this index (78). Thus, given the strong correlation between inflammatory markers and %BF in adolescents, the importance of assessing body fat composition is reiterated as a form of early intervention useful for acquiring and maintaining current and future health.

### Strengths and limitations

As strengths, it is believed that this is the first study to present a review on the subject specifically for adolescents, highlighting the novelty of this study in systematizing, through meta-analysis, the correlation of inflammatory markers with the percentage of body fat in these individuals. In addition, the studies included in this review were conducted with different populations, in 12 countries on different continents, evidencing the consistency of the data. Furthermore, the PRISMA Guideline was strictly followed to conduct this study and the Joanna Briggs Institute assessment tool confirmed the low risk of bias of the studies selected to compose the review. This study had limitations. There was no standardization in the method of assessing body fat composition, considering different procedures. All methods of body composition assessment were included, in order to broaden the inclusion of articles, due to the high heterogeneity in the choice of methods in already published studies. Body fat assessed by DEXA was shown to have a higher correlation with the markers when compared to another method. DEXA is considered the gold standard in body composition assessment and therefore has higher accuracy in this estimation. However, it is an expensive method, and not very accessible in the clinical setting, which makes simpler methods, such as bioimpedance, practical options that can, in controlled situations, provide results very close to those provided by the gold standard. Similarly, anthropometric measures that estimate body composition, especially skinfolds, are viable options for this assessment if trained assessors are available. The cross-sectional nature of most studies implies the need for longitudinal analyses, since it does not allow verifying causal relationships and does not consider changes in the concentration of markers due to numerous factors over time.

## Conclusion

This meta-analysis showed a significant correlation between high levels of leptin and CRP, and low levels of adiponectin, with body fat percentage in adolescents of both sexes. The Studies includes also showed that high levels of IL-6 and uric acid are associated with excess adiposity in these individuals. This highlights the need to routinely assess the body fat composition of adolescents, in order to identify those who have excess body fat, since they may present, at this stage of life, with chronic inflammation. This, in turn, may be the basis for the development of cardiometabolic diseases in adolescence, with maintenance or worsening in adulthood. This is especially important in the context of public health, in order to direct disease prevention and health promotion actions.

## Data Availability

The original contributions presented in the study are included in the article/Supplementary material, further inquiries can be directed to the corresponding author.
