# Supplementary material for: Leptin, CRP, and adiponectin correlate with body fat percentage in adolescents: systematic review and meta-analysis
Source: Front Nutr. 2025 Jul 11;12:1560080. doi: 10.3389/fnut.2025.1560080 (PMC12289669; doi:10.3389/fnut.2025.1560080)
Supplement: Supplementary file 1 [file Data_Sheet_1.docx]

**Appendix I –** Search Terms for Review.

| **SEARCH** | **STRATEGY BASE** |
| --- | --- |
| PUBMED | ("adolescent"[MeSH Terms] OR "adolescents"[Title/Abstract] OR "Adolescence"[Title/Abstract] OR "teen"[Title/Abstract] OR "teenager"[Title/Abstract] OR "youth"[Title/Abstract]) AND ("body composition"[MeSH Terms] OR "body compositions"[Title/Abstract] OR "body fat distribution"[MeSH Terms] OR "body fat patterning"[Title/Abstract] OR "adipose tissue"[MeSH Terms] OR "abdominal fat"[MeSH Terms] OR "body fat"[Title/Abstract] OR "abdominal fat"[Title/Abstract] OR "subcutaneous fat"[MeSH Terms] OR "adiposity"[MeSH Terms] OR "intra abdominal fat"[MeSH Terms] OR "visceral fat"[Title/Abstract] OR "subcutaneous fat, abdominal"[MeSH Terms]) AND ("cytokines"[MeSH Terms] OR "c reactive protein"[MeSH Terms] OR "hsCRP"[Title/Abstract] OR "high sensitivity C-reactive protein"[Title/Abstract] OR "leptin"[MeSH Terms] OR "interleukins"[MeSH Terms] OR "adiponectin"[MeSH Terms] OR "tumor necrosis factor alpha"[MeSH Terms] OR "inflammation"[Title/Abstract] OR "inflammatory markers"[Title/Abstract] OR "inflammatory biomarkers"[Title/Abstract]) |
| SCOPUS | ( TITLE-ABS-KEY ( adolescent )  OR  TITLE-ABS-KEY ( adolescence )  OR  TITLE-ABS-KEY ( teenager )  AND  TITLE-ABS-KEY ( body  AND fat )  OR  TITLE-ABS-KEY ( body  AND fat  AND percentage )  OR  TITLE-ABS-KEY ( body  AND fat  AND distribuition )  AND  TITLE-ABS-KEY ( cytokines )  OR  TITLE-ABS-KEY ( c  AND reactive  AND protein )  OR  TITLE-ABS-KEY ( leptin )  OR  TITLE-ABS-KEY ( interleukins )  OR  TITLE-ABS-KEY ( tumor  AND necrosis  AND factor  AND alpha )  OR  TITLE-ABS-KEY ( adiponectin )  OR  TITLE-ABS-KEY ( acid  AND uric )  OR  TITLE-ABS-KEY ( inflammatory  AND markers ) ) |
| COCHRANE | "adolescent" OR "adolescents" OR "Adolescence" OR "teen" OR "teenager" OR "youth" in Title Abstract Keyword AND "body composition" OR "body compositions" OR "body fat distribution" OR "body fat patterning" OR "adipose tissue" OR "abdominal fat" OR "body fat" OR "abdominal fat" OR "subcutaneous fat" OR "adiposity" OR "intra abdominal fat" OR "visceral fat" OR "subcutaneous fat, abdominal" in Title Abstract Keyword AND "cytokines" OR "c reactive protein" OR "hsCRP" OR "high sensitivity C-reactive protein" OR "leptin" OR "interleukins" OR "adiponectin" OR "tumor necrosis factor alpha" OR "uric acid" OR "inflammation" OR "inflammatory markers" OR "inflammatory biomarkers" in Title Abstract Keyword - in Trials (Word variations have been searched) |
| EMBASE | ('adolescent':ab,ti OR 'adolescents':ab,ti OR 'adolescence':ab,ti OR 'teen':ab,ti OR 'teenager':ab,ti OR 'youth':ab,ti) AND ('body composition':ab,ti OR 'body compositions':ab,ti OR 'body fat distribution':ab,ti OR 'body fat patterning':ab,ti OR 'adipose tissue':ab,ti OR 'body fat':ab,ti OR 'abdominal fat':ab,ti OR 'subcutaneous fat':ab,ti OR 'adiposity':ab,ti OR 'intra abdominal fat':ab,ti OR 'visceral fat':ab,ti OR 'subcutaneous fat, abdominal':ab,ti) AND ('cytokines':ab,ti OR 'c reactive protein':ab,ti OR 'hscrp':ab,ti OR 'high sensitivity c-reactive protein':ab,ti OR 'leptin':ab,ti OR 'interleukins':ab,ti OR 'adiponectin':ab,ti OR 'tumor necrosis factor alpha':ab,ti OR 'uric acid':ab,ti OR 'inflammation':ab,ti OR 'inflammatory markers':ab,ti OR 'inflammatory biomarkers':ab,ti) |
| SCHOLAR GOOGLE | ("adolescent" OR "adolescents" OR "Adolescence" OR "teen" OR "teenager") AND ("body composition" OR "body compositions" OR "adipose tissue" OR "body fat" AND "cytokines" OR "c reactive protein" OR "hsCRP" OR "high sensitivity C-reactive protein" OR "leptin" OR "interleukins" OR "adiponectin" OR "tumor necrosis factor alpha" OR "uric acid" OR "inflammation" OR "inflammatory markers" OR "inflammatory biomarkers") |
| PROQUEST | ("adolescent" OR "adolescents" OR "Adolescence" OR "teen" OR "teenager") AND ("body composition" OR "body compositions" OR "adipose tissue" OR "body fat" AND "cytokines" OR "c reactive protein" OR "hsCRP" OR "high sensitivity C-reactive protein" OR "leptin" OR "interleukins" OR "adiponectin" OR "tumor necrosis factor alpha" OR "uric acid" OR "inflammation" OR "inflammatory markers" OR "inflammatory biomarkers") |

**Appendix II**

**A.** Risk of bias for each individual study assessed by the Joanna Briggs Institute Critical Assessment Checklist for Cross-sectional Studies.

| **Studies** | **Criteria** | | | | | | | |
| --- | --- | --- | --- | --- | --- | --- | --- | --- |
|  | 1* | 2* | 3* | 4* | 5* | 6* | 7* | 8* |
| Agostinis-Sobrinho et al. 2020^53^ | Y | Y | Y | Y | Y | Y | Y | Y |
| Arslanian et al., 1998^25^ | Y | N | Y | Y | Y | Y | Y | Y |
| Bragança et al., 2020^11^ | Y | Y | Y | Y | N | N | Y | Y |
| Brandão et al., 2003^27^ | Y | N | Y | Y | Y | Y | Y | Y |
| Bugge et al., 2012^50^ | Y | Y | Y | Y | Y | Y | Y | Y |
| Bundy et al., 2011^35^ | Y | N | Y | Y | N | N | Y | Y |
| Caballero et al., 2008^32^ | Y | Y | Y | Y | Y | Y | Y | Y |
| Coutinho et al., 2015^58^ | Y | Y | Y | Y | N | Y | Y | Y |
| Hinriksdóttir et al 2015^52^ | Y | Y | Y | Y | Y | Y | Y | Y |
| Huang et al., 2004^36^ (Adiponectin) | Y | N | Y | Y | Y | Y | Y | Y |
| Huang et al., 2004^37^ (Leptin) | N | N | Y | Y | N | Y | Y | Y |
| Karen et al., 2010^34^ | N | N | Y | Y | Y | Y | Y | Y |
| Kelishadi et al., 2007^43^ | N | Y | Y | Y | Y | Y | Y | Y |
| Kruger; Pretorius; Schutte, 2010^48^ | Y | N | Y | Y | N | Y | Y | Y |
| Kuo et al., 2021^39^ | Y | Y | Y | Y | Y | Y | Y | Y |
| McVean et al., 2009^33^ | N | N | Y | Y | Y | Y | Y | Y |
| Menezes et al., 2018^29^ | Y | Y | Y | Y | Y | Y | Y | Y |
| Miranda et al., 2020^30^ | Y | Y | Y | Y | Y | Y | Y | Y |
| Mirhosseini et al., 2012^44^ | Y | Y | Y | Y | Y | Y | Y | Y |
| Plonka et al., 2011^47^ | N | N | Y | Y | Y | N | Y | Y |
| Schoppen et al., 2010^42^ | Y | N | Y | Y | Y | N | Y | Y |
| Serrano et al., 2010^9^ | Y | Y | Y | Y | Y | N | Y | Y |
| Sodré et al., 2020^31^ | N | N | Y | Y | Y | N | Y | Y |
| Stylianou et al., 2007^45^ | Y | N | Y | Y | Y | N | Y | Y |
| Vikram et al., 2004^40^ | Y | N | Y | Y | Y | Y | Y | Y |
| Zhang et al., 2022^26^ | N | N | Y | Y | Y | N | Y | Y |
| Warnberg et al., 2006^41^ | Y | N | Y | Y | Y | Y | Y | Y |
| Wu et al., 2015^38^ | Y | Y | Y | Y | Y | Y | Y | Y |
| Zeelie; Moss; Kruger, 2010^49^ | Y | Y | Y | Y | Y | Y | Y | Y |

Y: Yes; N: No.

1* Criteria for inclusion in the sample cleary defined

2* Study subjects and the setting described in detail

3* Exposure measured in a valid and reliable way

4* Objective and standard criteria for measurement

5* Confounding factors identified

6* Strategies to deal with confounding factors

7* Outcomes measured in a valid and reliable way

8* Appropriate statical analisys

**B.** Risk of bias for each individual study assessed by the Joanna Briggs Institute Critical Assessment Checklist for Cohort Studies.

| **Studies** | **Criteria** | | | | | | | | | | |
| --- | --- | --- | --- | --- | --- | --- | --- | --- | --- | --- | --- |
|  | 1* | 2* | 3* | 4* | 5* | 6* | 7* | 8* | 9* | 10* | 11* |
| Blogoswska et al., 2005^46^ | Y | Y | Y | N | N | Y | Y | Y | Y | NA | Y |
| Wen et al., 2014^51^ | Y | Y | Y | Y | Y | U | Y | Y | Y | Y | Y |

Y: Yes; N: No; NA: Not Applicable; U: Unclear.

1* Similar groups recruited from the same population

2* Exposures in a similar way to designate as persons to exposed and unexposed groups

3* Validly and reliably measured exposure

4* Identification factors

5* Strategies for dealing with confounders

6* Groups were free/participating at baseline (or at time of exposure)

7* Intermediate results validly and reliably

8* Follow-up time was observed and sufficient for results to occur

9* Follow-up was complete and, if not, reasons for loss of follow-up were described and exploratory

10* Strategies were used to address incomplete follow-up

11* Appropriate statistics

**Appendix III -** Graph of influence of studies for Leptin (A), CRP (B) and Adiponectin (C).


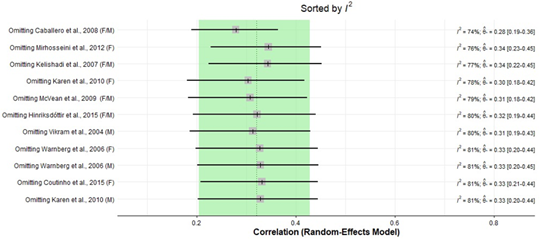


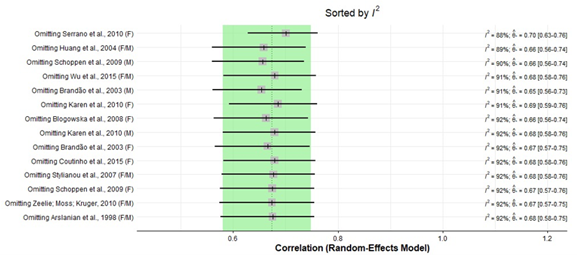


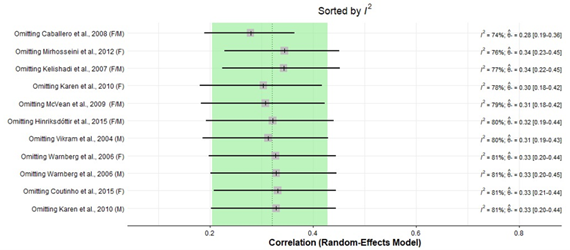


**Appendix IV -** Forestplot for correlation coefficients between body fat percentage and Leptin (A), CRP (B) in adolescents, according to the body fat assessment method.

**A**


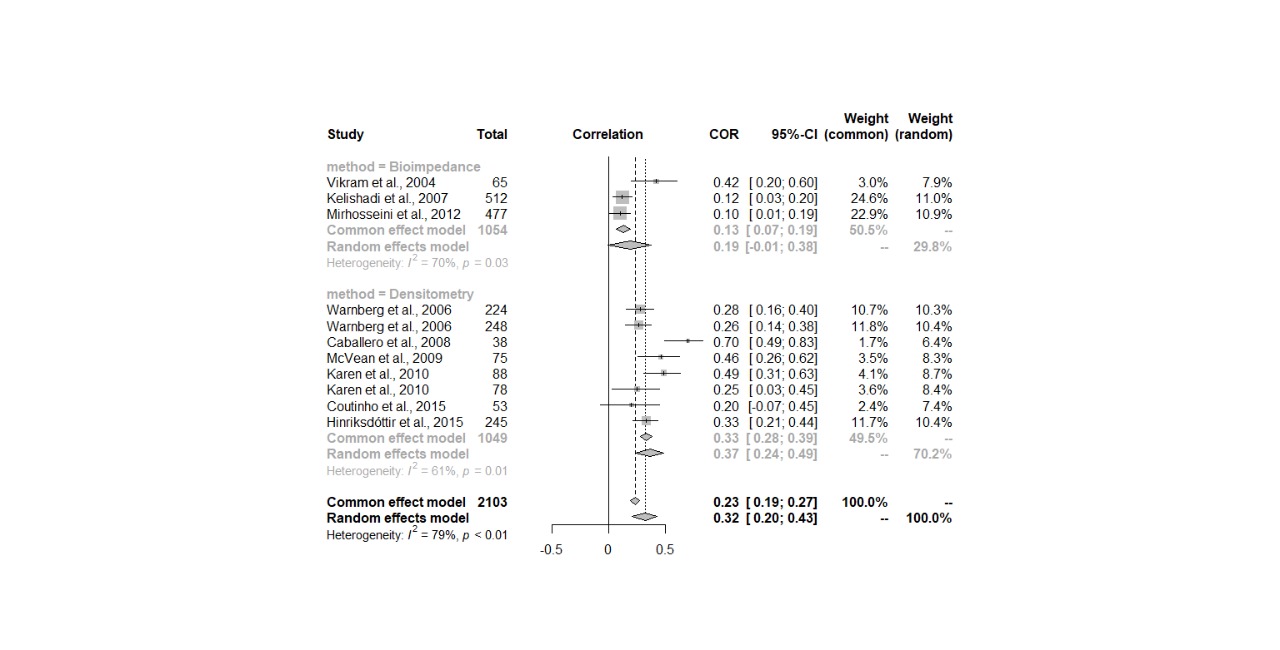


**B**


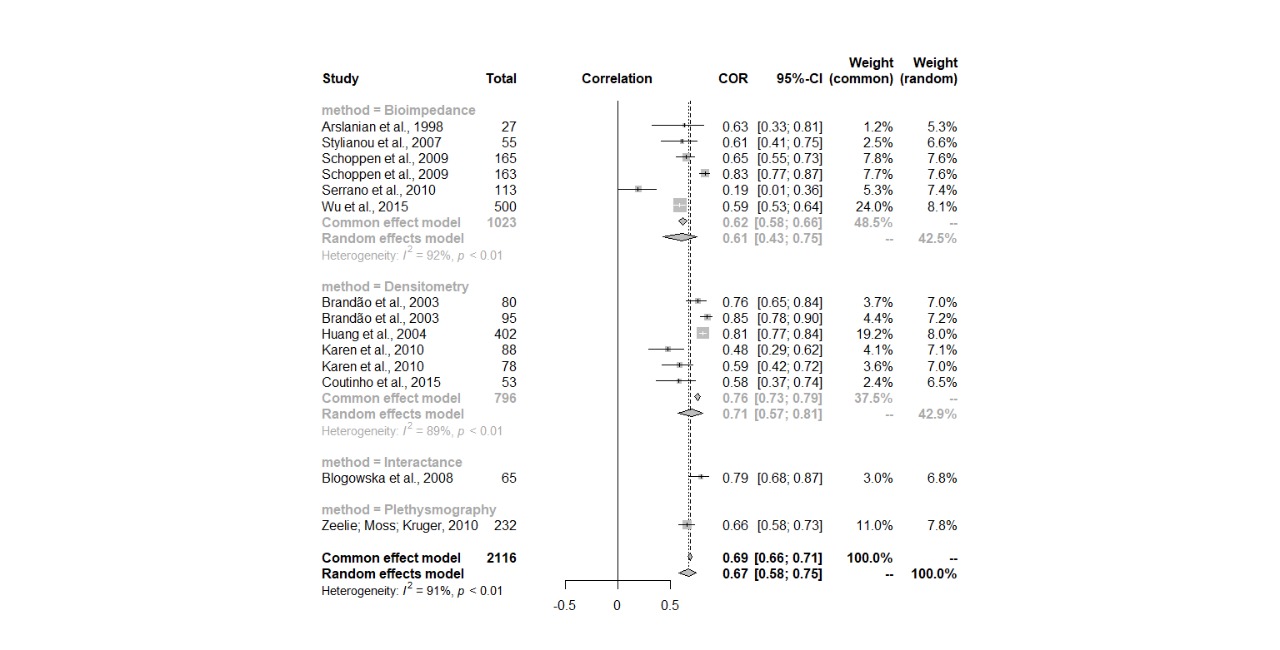


Legend:

This figure represents the individual effect of each study and the size indicates the sample weight.

The black line represents the Confidence Interval (CI) of each study.

The diamond systematizes the correlation between the inflammatory marker and the percentage of fat from all studies and the horizontal edges indicate the CI.

**Appendix V –** Forestplot for correlation coefficients between body-mass index and Leptin (A), CRP (B), Adiponectin (C) in adolescents.


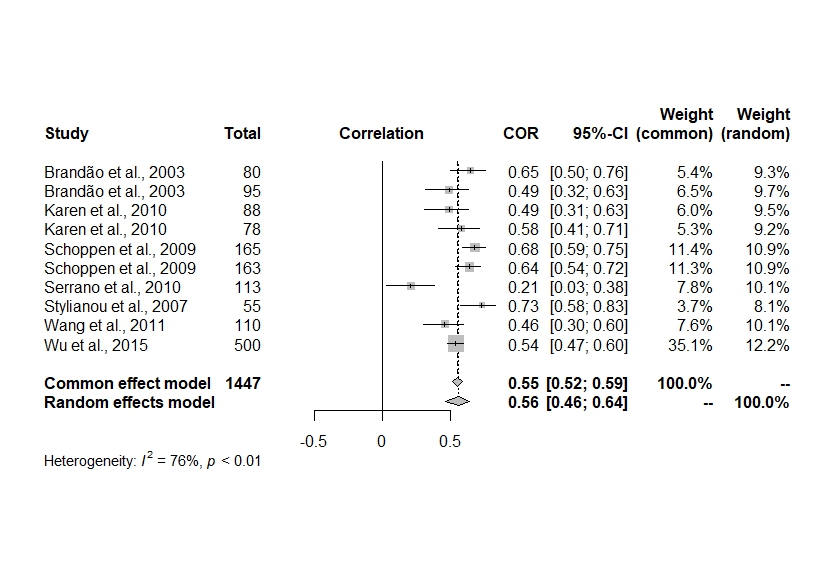


A


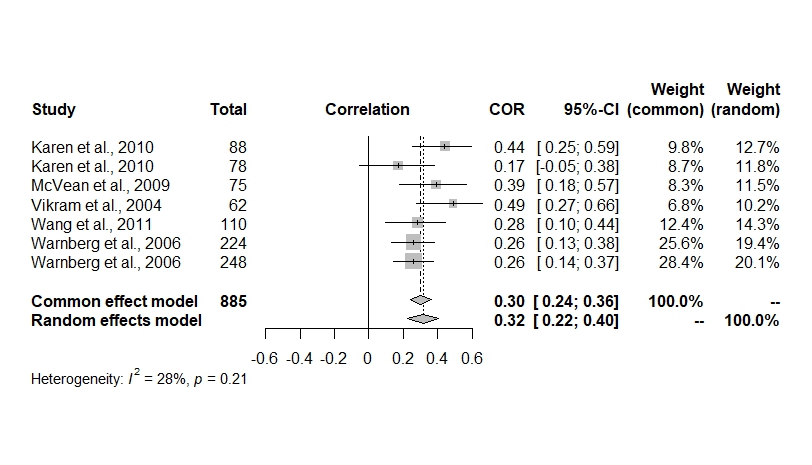


B

**
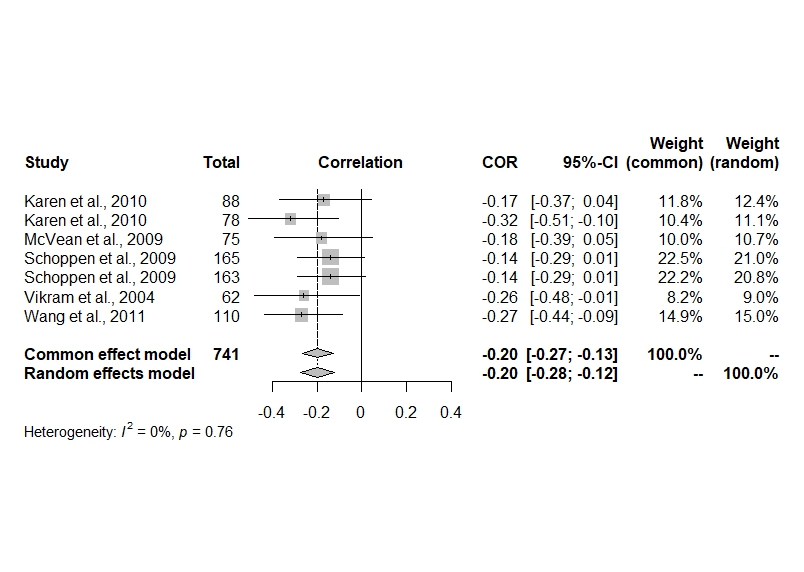
**

C

Legend:

This figure represents the individual effect of each study and the size indicates the sample weight.

The black line represents the Confidence Interval (CI) of each study.

The diamond systematizes the correlation between the inflammatory marker and the body-mass index from all studies and the horizontal edges indicate the CI.

**Appendix VI -** AMSTAR: A critical appraisal tool for systematic reviews that include randomized or non-randomised studies of healthcare interventions, or both.

| **1. Did the research questions and inclusion criteria for the review include the components of PICO?** | | | | | | | | | |  |  |  |
| --- | --- | --- | --- | --- | --- | --- | --- | --- | --- | --- | --- | --- |
| For Yes:  x  Population  x  Intervention  x  Comparator group  x  Outcome | | | Optional (recommended)  Timeframe for follow-up | | | x | | Yes No |  |  |  |  |
| **2. Did the report of the review contain an explicit statement that the review methods were established prior to the conduct of the review and did the report justify any significant deviations from the protocol?** | | | | | | | | | |  |  |  |
|  | For Partial Yes:  The authors state that they had a written protocol or guide that included ALL the following:  x  review question(s)  x  a search strategy  x  inclusion/exclusion criteria  x  a risk of bias assessment | | For Yes:  As for partial yes, plus the protocol should be registered and should also have specified:  a meta-analysis/synthesis plan, if appropriate, *and*  a plan for investigating causes of heterogeneity  justification for any deviations from the protocol | | | x | | Yes Partial Yes No |  |  |  |  |
| **3. Did the review authors explain their selection of the study designs for inclusion in the review?** | | | | | | | | | |  |  |  |
|  | For Yes, the review should satisfy ONE of the following:  *Explanation for* including only RCTs  x  OR *Explanation for* including only NRSI  OR *Explanation for* including both RCTs and NRSI | | | | | x | | Yes No |  |  |  |  |
| **4. Did the review authors use a comprehensive literature search strategy?** | | | | | | | | | |  |  |  |
|  | For Partial Yes (all the following): | | For Yes, should also have (all the following):  x  searched the reference lists / bibliographies of included studies  searched trial/study registries  included/consulted content experts in the field  x  where relevant, searched for grey literature  conducted search within 24 months of completion of the review | | |  | |  |  |  |  |  |
|  | searched at least 2 databases (relevant to research question)  x  provided key word and/or search strategy  x  x  justified publication restrictions | |  |  |  | x | | Yes Partial Yes No |  |  |  |  |
|  | (e.g. language) | |  |  |  |  | |  |  |  |  |  |
|  | **5. Did the review authors perform study selection in duplicate?** | | | | |  | | |  |  |  |  |
|  | For Yes, either ONE of the following:  x  at least two reviewers independently agreed on selection of eligible studies and achieved consensus on which studies to include  OR two reviewers selected a sample of eligible studies and achieved good agreement (at least 80 percent), with the remainder selected by one reviewer. | | | | | x | | Yes No |  |  |  |  |
| **6. Did the review authors perform data extraction in duplicate?** | | | | | | | | | | | | |
| For Yes, either ONE of the following:  x  at least two reviewers achieved consensus on which data to extract from included studies  OR two reviewers extracted data from a sample of eligible studies and achieved good agreement (at least 80 percent), with the remainder extracted by one reviewer. | | | | | | | x  Yes  No | | | | | |
| **7. Did the review authors provide a list of excluded studies and justify the exclusions?** | | | | | | | | | | | | |
|  | | For Partial Yes:  x  provided a list of all potentially relevant studies that were read  in full-text form but excluded from the review | | For Yes, must also have:  Justified the exclusion from the review of each potentially relevant study | | | Yes  x  Partial Yes  No | | | | | |
| **8. Did the review authors describe the included studies in adequate detail?** | | | | | | | | | | | | |
|  | | For Partial Yes (ALL the following):  x  described populations  x  described interventions  x  described comparators  x  described outcomes  x  described research designs | | For Yes, should also have ALL the following:  described population in detail  described intervention in detail (including doses where relevant)  described comparator in detail (including doses where relevant)  described study’s setting  timeframe for follow-up | | | Yes  x  Partial Yes  No | | | | | |
| **9. Did the review authors use a satisfactory technique for assessing the risk of bias (RoB) in individual studies that were included in the review?** | | | | | | | | | | | | |
|  | | **RCTs**  For Partial Yes, must have assessed RoB from  unconcealed allocation, *and*  lack of blinding of patients and assessors when assessing outcomes (unnecessary for objective outcomes such as all-  cause mortality) | | For Yes, must also have assessed RoB from:  allocation sequence that was not truly random, *and*  selection of the reported result from among multiple measurements or analyses of a specified outcome | | | Yes  Partial Yes  No  x  Includes only NRSI | | | | | |
|  | | **NRSI**  For Partial Yes, must have assessed RoB:  from confounding, *and*  from selection bias  **10. Did the review authors report o** | | For Yes, must also have assessed RoB:  x  methods used to ascertain exposures and outcomes, *and*  x  selection of the reported result from among multiple measurements or analyses of a specified outcome  **n the sources of funding for the studies inc** | | | Yes  x  Partial Yes  No  Includes only RCTs  **luded in the review?** | | | | | |
|  | | For Yes  x  x  Must have reported on the sources of funding for individual studies included Yes in the review. Note: Reporting that the reviewers looked for this information No but it was not reported by study authors also qualifies | | | | | | | | | | |
| **11. If meta-analysis was performed did the review authors use appropriate methods for statistical combination of results?** | | | | | | | | | | | | |
|  | **RCTs**  For Yes:  The authors justified combining the data in a meta-analysis  AND they used an appropriate weighted technique to combine study results and adjusted for heterogeneity if present.  AND investigated the causes of any heterogeneity | | | | | Yes  No  x  No meta-analysis conducted | | | | |  | |
|  | **For NRSI**  For Yes:  The authors justified combining the data in a meta-analysis  AND they used an appropriate weighted technique to combine study results, adjusting for heterogeneity if present  AND they statistically combined effect estimates from NRSI that were adjusted for confounding, rather than combining raw data, or justified combining raw data when adjusted effect estimates were not available  AND they reported separate summary estimates for RCTs and NRSI separately when both were included in the review | | | | | Yes  No  x  No meta-analysis conducted | | | | |  | |
| **12. If meta-analysis was performed, did the review authors assess the potential impact of RoB in individual studies on the results of the meta-analysis or other evidence synthesis?** | | | | | | | | | | | | |
|  | For Yes:  included only low risk of bias RCTs  OR, if the pooled estimate was based on RCTs and/or NRSI at variable RoB, the authors performed analyses to investigate possible impact of RoB on summary estimates of effect. | | | | | Yes  No  x  No meta-analysis conducted | | | | |  | |
| **13. Did the review authors account for RoB in individual studies when interpreting/ discussing the results of the review?** | | | | | | | | | | | | |
|  | For Yes:  included only low risk of bias RCTs  x  OR, if RCTs with moderate or high RoB, or NRSI were included the review provided a discussion of the likely impact of RoB on the results | | | | | x  Yes  No | | | | |  | |
| **14. Did the review authors provide a satisfactory explanation for, and discussion of, any heterogeneity observed in the results of the review?** | | | | | | | | | | | | |
|  | For Yes:  x  There was no significant heterogeneity in the results  OR if heterogeneity was present the authors performed an investigation of sources of any heterogeneity in the results and discussed the impact of this on the results of the review | | | | | Yes  x  No | | | | |  | |
| **15. If they performed quantitative synthesis did the review authors carry out an adequate investigation of publication bias (small study bias) and discuss its likely impact on the results of the review?** | | | | | | | | | | | | |
|  | For Yes:  performed graphical or statistical tests for publication bias and discussed the likelihood and magnitude of impact of publication bias | | | | | Yes  No  x  No meta-analysis conducted | | | | |  | |
| **16. Did the review authors report any potential sources of conflict of interest, including any funding they received for conducting the review?** | | | | | | | | | | | | |
|  | For Yes:  x  The authors reported no competing interests OR  The authors described their funding sources and how they managed potential conflicts of interest | | | | x  Yes  No | | | | | | |  |
